# Unmasking Retinitis Pigmentosa complex cases by a whole genome sequencing algorithm based on open-access tools: hidden recessive inheritance and potential oligogenic variants

**DOI:** 10.1186/s12967-020-02258-3

**Published:** 2020-02-12

**Authors:** María González-del Pozo, Elena Fernández-Suárez, Marta Martín-Sánchez, Nereida Bravo-Gil, Cristina Méndez-Vidal, Enrique Rodríguez-de la Rúa, Salud Borrego, Guillermo Antiñolo

**Affiliations:** 1 Department of Maternofetal Medicine, Genetics and Reproduction, Institute of Biomedicine of Seville, University Hospital Virgen del Rocío/CSIC/University of Seville, Avenida Manuel Siurot s/n, 41013 Seville, Spain; 2 Centro de Investigación Biomédica en Red de Enfermedades Raras (CIBERER), Seville, Spain; 3 Department of Ophthalmology, University Hospital Virgen Macarena, Seville, Spain; 4 ReticsPatologia Ocular, OFTARED, Instituto de Salud Carlos III, Madrid, Spain

**Keywords:** Retinitis Pigmentosa, Inherited retinal dystrophies, WGS, *USH2A*, *ADGRV1*, *PDZD7*, NGS

## Abstract

**Background:**

Retinitis Pigmentosa (RP) is a clinically and genetically heterogeneous disorder that results in inherited blindness. Despite the large number of genes identified, only ~ 60% of cases receive a genetic diagnosis using targeted-sequencing. The aim of this study was to design a whole genome sequencing (WGS) based approach to increase the diagnostic yield of complex Retinitis Pigmentosa cases.

**Methods:**

WGS was conducted in three family members, belonging to one large apparent autosomal dominant RP family that remained unsolved by previous studies, using Illumina TruSeq library preparation kit and Illumina HiSeq X platform. Variant annotation, filtering and prioritization were performed using a number of open-access tools and public databases. Sanger sequencing of candidate variants was conducted in the extended family members.

**Results:**

We have developed and optimized an algorithm, based on the combination of different open-access tools, for variant prioritization of WGS data which allowed us to reduce significantly the number of likely causative variants pending to be manually assessed and segregated. Following this algorithm, four heterozygous variants in one autosomal recessive gene (*USH2A*) were identified, segregating in pairs in the affected members. Additionally, two pathogenic alleles in *ADGRV1* and *PDZD7* could be contributing to the phenotype in one patient.

**Conclusions:**

The optimization of a diagnostic algorithm for WGS data analysis, accompanied by a hypothesis-free approach, have allowed us to unmask the genetic cause of the disease in one large RP family, as well as to reassign its inheritance pattern which implies differences in the clinical management of these cases. These results contribute to increasing the number of cases with apparently dominant inheritance that carry causal mutations in recessive genes, as well as the possible involvement of various genes in the pathogenesis of RP in one patient. Moreover, our WGS-analysis approach, based on open-access tools, can easily be implemented by other researchers and clinicians to improve the diagnostic yield of additional patients with inherited retinal dystrophies.

## Background

Retinitis Pigmentosa (RP, ORPHA:791) is the most common form of inherited retinal dystrophies (IRD), affecting 1 in 4000 individuals worldwide [1]. RP is characterized by the primary death of rods, which typically manifests with progressive night blindness followed by visual field constriction. As the disease progresses, cones dysfunction also occurs, leading to decreased visual acuity and central vision loss [2]. RP is defined by a huge phenotypic variability, in which age of onset and disease progression can vary from patient to patient, even within the same family (inter- and intra-familial variability) [3, 4]. Moreover, RP is one of the most genetically heterogeneous disorders, as mutations in 88 genes have been associated so far [5]. RP can be inherited as an autosomal dominant (adRP), autosomal recessive (arRP) or X-linked (XLRP) trait. However, in a large percentage of cases, the mode of inheritance is unknown due to the absence of additional affected members (simplex RP, sRP) [6, 7]. In other cases, the mode of inheritance can be inaccurately assumed due to pseudo-dominance of certain XLRP variants [8, 9], or the presence of more than one genetic causes in the same family [10–12].

In this scenario, receiving a genetic diagnosis becomes increasingly important to confirm the clinical diagnosis [13], to provide genetic and reproductive counseling for a proper clinical management of patients [14] and due to the development of gene therapy for some retinal dystrophies [15]. The methods and tools available for genetic diagnosis have evolved dramatically during the last two decades. Nowadays, different next-generation sequencing (NGS) approaches are commonly conducted for the genetic diagnosis of IRD, most of them based on targeted sequencing of a variable number of disease-associated genes [16–19]. The overall diagnostic yield of targeted sequencing is 55–65% [7, 20, 21], suggesting the implication of both novel genes or mutations not detectable or filtered by standard diagnostic algorithms such as structural, deep-intronic, non-coding or synonymous variants [22]. Whole genome sequencing (WGS) has been shown to overcome some of the disadvantages of whole exome sequencing (WES) and targeted gene panels, due to WGS coverage uniformity [23], allowing also the identification of deep-intronic variants and structural variations. Indeed, a comparative study between WGS and targeted gene panels concluded that WGS may improve the pathogenic variant detection rate by facilitating detection of structural variations and variants in regulatory regions [24]. The remaining challenge will be in handling the large amount of data generated and variant interpretation, which is further aggravated by the absence of consensus workflows. Thus, WGS could be useful to characterize those cases in which the screening for previously identified disease-causing variants had been inconclusive.

The aim of this work was to uncover the genetic cause of RP in one Spanish family using WGS. The causal mutations underlying the phenotype of this family were not previously identified using gene-panel sequencing. Therefore, we have implemented and optimized a diagnostic algorithm for the systematic analysis of WGS data, which included variant annotation, filtering, prioritization, bioinformatics pathogenicity predictions, Sanger sequencing validation and segregation studies.

## Methods

### Subjects, clinical evaluation and previous studies

**Fig. 1:**
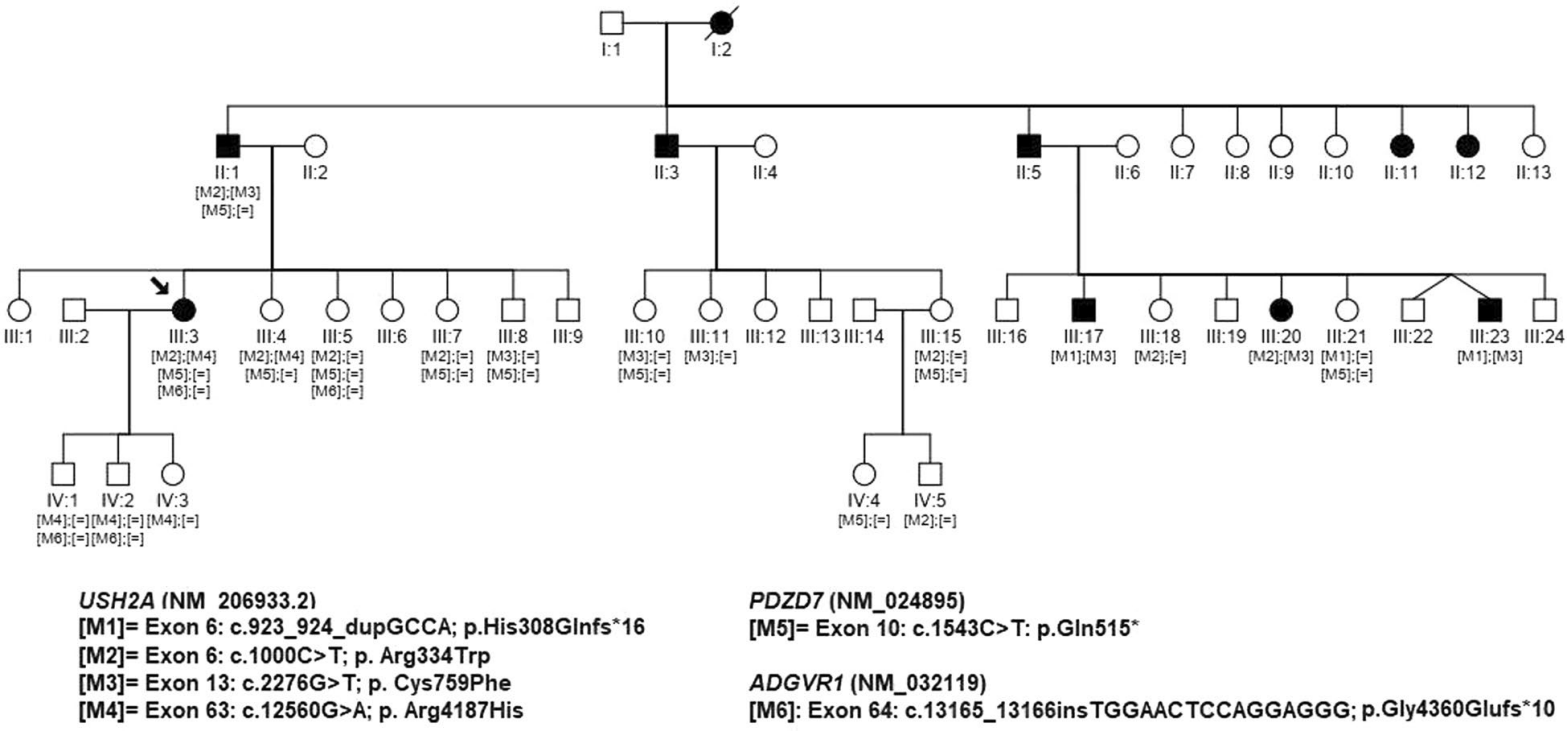
Pedigree of the RP family and segregation analysis studies. Below the individuals, genotypes are presented for each variant segregating with RP. Index patients are indicated with a black arrow. [M] represents identified mutations; [=] indicates wild-type alleles

Nineteen participants from the same family, including five affected individuals, were recruited and received comprehensive ophthalmic evaluations. Three individuals underwent WGS (III:3, III:23 and IV:1). The DNA of these three individuals together with the DNA of sixteen additional family members were used to make the segregation analysis of candidate variants by Sanger sequencing (II:1, III:3, III:4, III:5, III:7,III:8, III:10, III:11, III:15, III:17, III:18, III:20, III:21, III:23, IV:1, IV:2, IV:3, IV:4 and IV:35) (Fig. 1).

Clinical diagnosis of retinal dystrophy was based on fundus examination, visual acuity, computerized testing of central and peripheral visual fields and electroretinography (ERG) findings. RP was defined as bilateral visual loss, initial night blindness, restrictions of visual field, gradual increased bone spicule pigmentation, decrease of visual acuity, attenuation of retinal vessels and reduced or undetectable ERG [2].

Peripheral blood was collected from all subjects to extract genomic DNA using standard protocols. Prior to WGS, individual III:3 was analyzed by targeted sequencing using a panel of 64 IRD genes [16] without achieving a genetic diagnosis.

Moreover, in order to facilitate the filtering and prioritization steps during the bioinformatic analysis, an in-house database containing WGS data was used as pseudo-controls. This pseudo-control cohort was composed of six unaffected individuals belonging to unrelated IRD families processed under similar conditions.

### Whole genome sequencing and data analysis

WGS has been performed by Edinburgh Genomics using Illumina SeqLab, which integrates Illumina TruSeq library preparation, Illumina cBot2 cluster generation, Illumina HiseqX sequencing, Hamilton Microlab STAR integrative automation, and Genologics Clarity LIMS X Edition.

Briefly, genomic DNA (gDNA) samples with a concentration of 20–100 ng/µl were sheared to a 450 bp mean insert size using a Covaris LE220 focused-ultrasonicator. The inserts were blunt ended, A-tailed, size selected and the TruSeq adapters were ligated onto the ends. The insert size for each library was evaluated using the Caliper GX Touch to ensure that the mean fragment sizes fell between 300 and 800 bp. The concentration of each library was calculated using a Roche LightCycler 480 and a Kapa Illumina Library Quantification kit to ensure that the concentration of each library was between 1.1 and 8 nM.

The libraries were normalized to 1.5 nM and were denatured for clustering and sequencing at 300 pM using Hamilton MicroLab STAR with Genologics Clarity LIMS X (4.2) Edition. Libraries were clustered onto a HiSeq X Flow cell v 2.5 on cBot2s and the clustered flow cell was transferred to a HiSeqX platform for sequencing using a HiSeqX Ten Reagent kit v2.5.

**Fig. 2:**
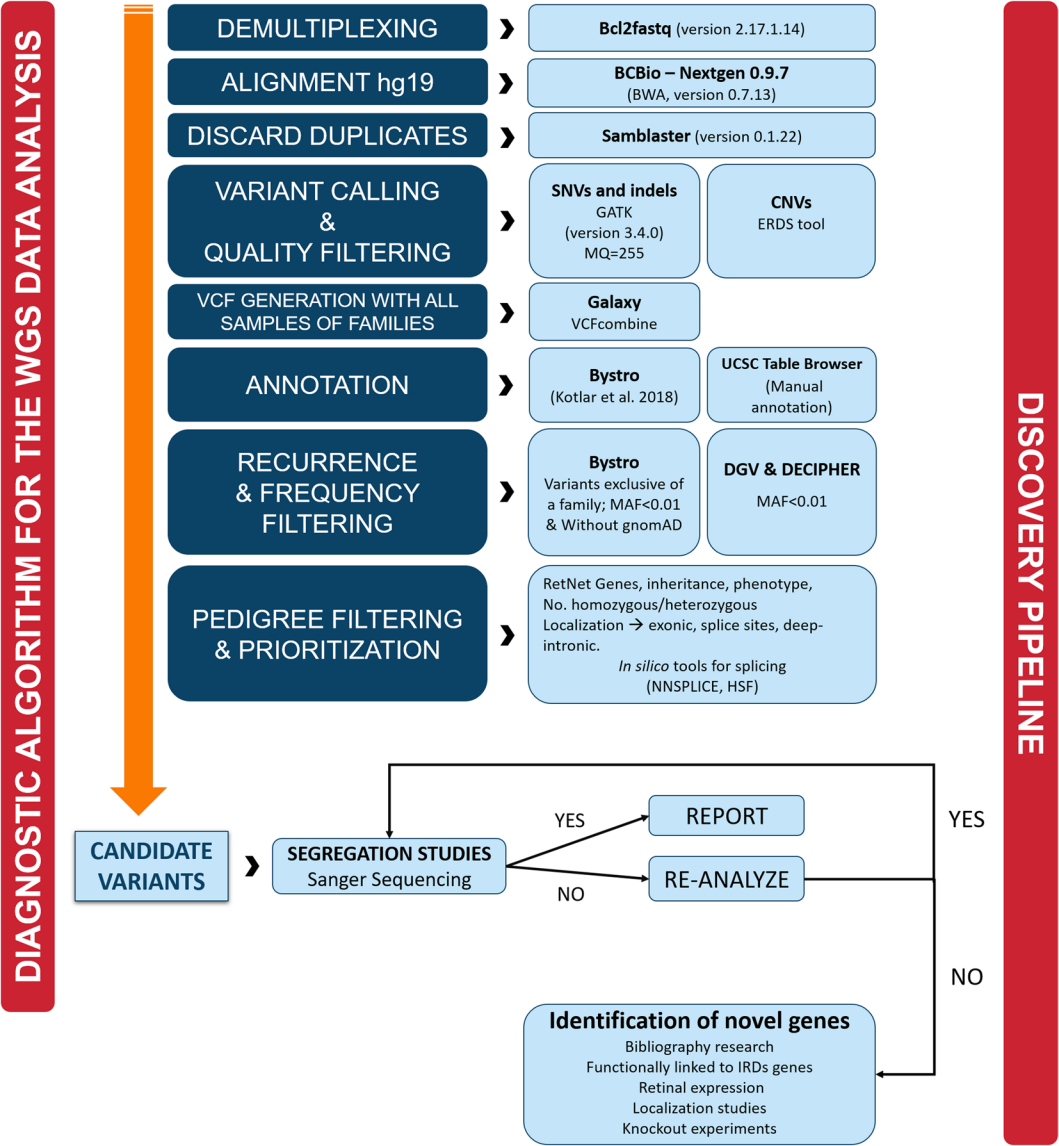
Pipeline design for WGS data analysis. Bioinformatics analysis including mapping, calling, filtering, and annotation of variants, followed by a pathogenicity analysis in which the candidate variants are prioritized and validated with the aim of finding the causal mutation and informing the patient. A reanalysis of the data is conducted when no candidate variants are identified in the first analysis. If no candidate variants are detected in any of the known IRD genes, causal mutations in novel genes are evaluated (discovery pipeline)

The developed algorithm for WGS data analysis is shown in Fig. 2. The bioinformatics analysis was executed using several bioinformatics tools: bcl2fastq v.2.17.1.14 (Illumina)for demultiplexing, allowing one mismatch when assigning reads to barcodes; BCBio-Nextgenv.0.9.7 (https://github.com/bcbio/bcbio-nextgen) to perform alignment [25], BAM file preparation and variant detection, BCBio uses BWA memv.0.7.13 [26] to align the raw reads to the human reference genome (hg19); samblaster v.0.1.22 [27] to mark the duplicated fragments and the Genome Analysis Toolkit (GATK v.3.4-0-g7e26428) [28] for indels realignment and base recalibration. Finally, the genotype likelihoods are calculated using GATK HaplotypeCaller (3.4-0-g7e26428) creating a final VCF file for each of the sequenced samples.

Additionally, in order to facilitate the subsequent data analysis, the tool VCF Combine, available in the Galaxy web-based platform [29], was used to generate a combined VCF file containing all variants from sequenced samples selected in each of the optimization phases of the algorithm.

CNVs analysis was conducted employing the tool Estimation by Read Depth with Single-nucleotide variants (ERDS) [30]. CNVs annotation was done using an in-house solution based on UCSC Table Browser [31]. Large deletions and duplications were visually inspected with Integrative Genomics Viewer (IGV). All likely pathogenic CNVs were checked in Database of Genomic Variants (DGV) [32] and DECIPHER [33].

### Variants filtering, prioritization and pathogenicity assessment

**Fig. 3:**
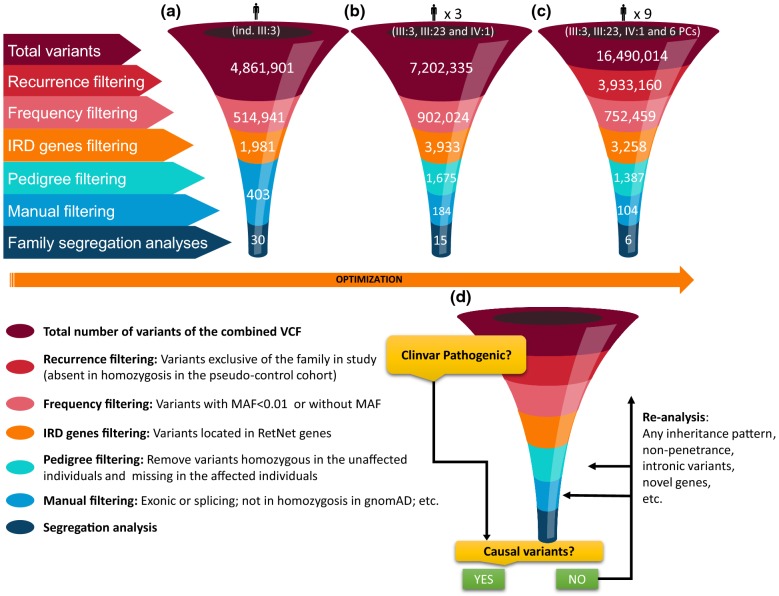
Optimization of tertitary WGS-data analysis. **a** The VCF from the index patient (individual III:3), was filtered by MAF, followed by an “IRD gene filtering” and a manual filtering. **b** The VCF from three individuals belonging to the same family (individuals III:3, III:23 and IV:1) allowed us to introduce a new filtering step based on the pedigree information (“pedigree filtering”) leading to a reduction in the number of variants pending to be interrogated manually. **c** The combined VCF from the three individuals sequenced and 6 additional unaffected individuals belonging to unrelated families was used as the pseudo-control cohort. This modification allowed us to introduce another filtering step (“recurrence filtering”) in which only variants exclusive of the family in study remained for further analysis. **d** Proposed algorithm for variant filtering and prioritization using WGS data of patients with inherited retinal dystrophy. Ind: individual; PCs: Pseudo-controls

The tertiary WGS data analysis was done following a step-by-step in-house algorithm, using the online tool Bystro [34] and a VCF file as starting point (Fig. 3). Subsequently, several filtering steps were applied: (i) the “recurrence filtering” applicable if a combined VCF file containing variants of all sequenced samples (including pseudo-controls) was available. This filter allows discarding sequencing artefacts and polymorphisms leaving only variants exclusive of the family under study and absent in homozygosis in the pseudo-control cohort. Prior to the application of this filter, we checked if there was any variant consistent with the patient’s phenotype, that is, variants previously associated with any type of IRD, described as pathogenic or as likely pathogenic in the ClinVar database. For this purpose, Bystro’s filters, such as, ‘ClinVar clinical significance’ and ‘ClinVar phenotype list’, were employed. Moreover, ‘conflicting interpretations of pathogenicity’ variants were also checked just in case they were conflicting between pathogenic/VUS. (ii) The “frequency filtering”, was used to discard variants with a MAF > 0.01 in the Genome Aggregation Database (gnomAD). (iii) The “IRD genes filtering”, to prioritize variants located in any of the 274 genes previously associated with IRD according to the RetNet [5] (Additional file 1: Table S1). This filter allows the prioritization of all those exonic and intronic variants in these genes, regardless of the distance from splice sites, before to evaluate candidate variants in novel genes. (iv) The “pedigree filtering”, help us to prioritize variants according to their zygosity and phenotype, as long as the starting point was not a single VCF file. This filter should be applied taking into account the specific pedigree of each family. In this case, heterozygous or homozygous variants in one patient were prioritized, whether or not they were in the other affected individual. Moreover, the homozygous variants in the unaffected individual were discarded. Furthermore, since WGS was conducted in the affected individual III:3 and in her unaffected son IV:1, all those heterozygous variants located in *cis* in the same gene could be filtered out.

Following this, a manual prioritization was conducted by which variants in the coding exons or in the ± 10 bp of their flanking intronic regions were prioritized. If no causal mutation were detected, deep-intronic variants were considered. Although only variants with MAF < 0.01 have been selected, the absence of homozygotes in gnomAD should be checked manually for each candidate variant. For intronic variants, three online tools were used to assess the impact on splicing mechanisms: two algorithms included in Human Splicing Finder (HSF [35] and MaxEntScan [36]) and NNSPLICE [37]. Specific thresholds were defined based on a known deep-intronic variants validation for two tools: a minimum score of 2 and score variation > 15% for MaxEnt and a minimum score of70 and score variation > 10% for HSF, were necessary to pass the quality threshold. For NNSPLICE, we use default settings (cut-off > 0.4) and a score difference between wild-type and mutated sequence > 10% to be considered for further analysis [38]. The pathogenicity prediction of variants was performed by SIFT [39], PolyPhen-2 [40] and/or Mutation Taster [41] depending on the type of mutation. Candidate variants were classified using the ACMG guidelines [42].

Sanger sequencing was conducted in order to validate and segregate all the candidate variants in available family members. Specifically gDNA from 19 individuals (Fig. 1) was used to verify segregation of the sequence alteration with the phenotype by conventional Sanger sequencing according to the manufacturer’s protocols (BigDye^®^ Terminator v3.1 Cycle Sequencing Kit, 3730 DNA Analyzer, Applied Biosystems, USA) (Primer sequences and reaction conditions are available upon request).

The nomenclature of all variants was adjusted to the Human Genome Variation Society guidelines using Mutalyzer [43].

## Results

### Clinical features

The analyzed family was of Spanish origin. Affected individuals received a well-defined clinical diagnosis of RP and had a suspected autosomal dominant pedigree due to the existence of multiple affected individuals of both genders in three consecutive generations (Fig. 1). Clinical findings of the sequenced patients are reported in Table 1.

**Table 1:** Clinical characteristics of the affected individuals analyzed by WGS

Pedigree subject	Onset age	First symptom	Age at time of the genetic assessment	Symptoms at time of the genetic assessment	Fundus examination	Clinical diagnosis	Other considerations
III:3 (female)	22	Night blindness	38	Night blindness; reduction of the visual field	N.A.	adRP → arRP	Affected father
III:23 (male)	33	Decreased of visual acuity	43	Concentric reduction of the visual field (5° central); decreased visual acuity	Narrowed vessels; bone splicule pigmentation; RPE degeneration	adRP → arRP	One older brother with the same clinical diagnosis (III:17); one older sister with a later onset age (III:20)

*adRP* autosomal dominant Retinitis Pigmentosa, *arRP* autosomal recessive Retinitis Pigmentosa, *N.A.* not available, *RPE* retinal pigment epithelium

### NGS data quality

Quality assurance and quality control are essential to ensure the reliability of the generated data. Genome sequencing in the three sequenced individuals produced an average mapping yield of 134.2 Gb ± 4.12 (mean ± SD) and an average coverage of 34.2x ± 0.97 (mean ± SD). The 99.4% of reads were mapped, and the duplicated reads percentage was 15.5%. The total bases Q ≥ 30 was 85.9%. The Q score of 30 to a base is equivalent to the probability of an incorrect base call 1 in 1000 times. This means that the base call accuracy is 99.9%, thus, all the reads will have zero errors and ambiguities in the 85.9% of the bases. All these parameters indicated that WGS data had a good quality for continuing the analysis.

### Diagnostic algorithm optimization for the WGS data analysis

Tertiary data analysis comprising filtering, annotation, prioritization and biological interpretation of candidate variants was performed using an in-house algorithm (Fig. 2). Tertiary data analysis is the most complex, experiment-specific, time-consuming and manual phase of the NGS data analysis pipeline, and therefore, part of this work implied the optimization of the filtering steps (Fig. 3).

Initially, an unprocessed VCF file containing all the variants present in one single individual (~ 4.8 millions) was used (Fig. 3a). After selecting all variants with a MAF < 0.01 located in IRD genes, around 400 variants remained for manual prioritization and in silico tools predictions. Of these, around 30 possibly causative variants were selected for family segregation studies by Sanger sequencing. As a first approach, VCFs for each individual were annotated using Bystro but this process hampered the integration of the data for the rest of individuals belonging to the same family, requiring considerable time and effort. Therefore, a second approach consisting in the generation of a single VCF file that combined the data of the three individuals belonging to the same family was followed (Fig. 3b). This allowed the integration of all the data automatically using Bystro filtering options, and taking into account the phenotype data of each individual (“pedigree filtering”). Although the initial number of variants was greater in this file (~ 7.2 millions), after applying the corresponding filters, the number of variants remaining for manual prioritization was significantly reduced (from 403 to 184) (Fig. 3b) but it was still excessive. Finally, to further optimize the data analysis, a single VCF file containing all the variants (~ 16.5 millions of variants) present in the 3 individuals of our family and the 6 individuals of the pseudo-control cohort was generated (Fig. 3c). The use of this single VCF file allowed us to filter out sequencing artefacts and polymorphisms which facilitated data interpretation. Interestingly, after applying the “recurrence filtering”, the number of variants exclusive of one family dropped from to ~ 7.2 millions to ~ 3.9 millions. This efficient strategy allowed us to further reduce the number of likely causative variants pending to be manually assessed and segregated (from 184 to 104) (Fig. 3c). Hence, the proposed algorithm for the variant prioritization of WGS data from IRD patients comprised the application of five different filters (Fig. 3d).

Likewise, if no candidate variants in any of the IRD genes segregated with the disease in the family, mutations in novel genes would be considered following the discovery pipeline (Fig. 2). The prioritization of variants in novel genes would be done considering multiples factors such as the pathogenicity predictors provided by Bystro (CADD [44], PhastCons [45] and PhyloP [46]), the absence of homozygotes in gnomAD, bibliography searching, the expression of the gene in retina available at public expression databases, the presence of retinal changes in knockout mouse models, the association of the novel protein in known retinal interaction networks (STRING [47]), etc.

### Identification of mutations by whole genome sequencing

The application of the diagnostic algorithm led to the initial identification of four candidate variants (M1–M4) in the analyzed family (Table 2). Among these, three had been previously reported as pathogenic or likely pathogenic variants in clinical databases: two missense (M2: p.(Arg334Trp) and M3: p.(Cys759Phe)) and one frameshift mutation (M1: p.(His308Glnfs*16) in *USH2A.* The last variant consisted in one missense variant in *USH2A* (M4: p.(Arg4187His)). A further analysis, led to the identification of two additional candidate variants (M5 and M6), comprising one nonsense variant in *PDZD7* (M5: p.(Gln515*)) and one novel frameshift variant in *ADGRV1* (M6: p.(Gly4360Glufs*10)). Interestingly, M4 and M5 variants have been reported in the general population (gnomAD) with a very low MAF, but no entry in clinical databases has been made for any of them (Table 2).

**Table 2:** Detailed information of the clinically relevant variants identified in the family

Gene	Status	Variations	MAF (GnomAD)	ClinVar	LOVD	SIFT	Poly-Phen2	Mutat taster	ACMG	dSNP ID
*USH2A*	HET	M1: c.923_924dupGCCA p. His308Glnfs*16	0.0000568 (16 alleles, 0 Hom)	P (ID: 48615)	P	_	_	_	5 (P)	rs397518043
*USH2A*	HET	M2: c.1000C>T p.Arg334Trp	0.0000531 (15 alleles, 0 Hom)	P/LP (ID: 228411)	P	_	_	_	5 (P)	rs397517963
*USH2A*	HET	M3: c.2276G>T p.Cys759Phe	0.0009677 (273 alleles, 0 Hom)	P/LP (ID: 2356)	P/LP	_	_	_	4 (LP)	rs80338902
*USH2A*	HET	M4: c.12560G>A p. Arg4187His	0.0000213 (6 alleles, 0 Hom)	N.R.	N.R.	D 100%	B (0.066)	SNP	3 (VUS)	rs147304271
*PDZD7*	HET	M5: c.1543C>T p.Gln515*	0.0000134 (2 alleles, 0 Hom)	N.R.	N.R.	D 100%	_	DC	5 (P)	rs979094623
*ADGRV1*	HET	M6: c.13165_13166 insTGGAA CTCCAGG AGGG p.Gly4360Glufs*10	_	N.R.	N.R.	_	PD (0.999)	DC	4 (LP)	_

Reference sequences used: *USH2A* NM_206933, *PDZD7* NM_024895 and *ADGRV1* NM_032119

*B* bening, *D* damaging, *DC* disease causing, *Hom* homozygous, *ID* identifier, *L.P.* likely pathogenic, *N.R.* not reported, *P* pathogenic, *PD* probably damaging, *SNP* single nucleotide polymorphism, *VUS* variant of unknown significance

Although the family in study was clinically diagnosed of RP with presumed autosomal dominant inheritance, our diagnostic algorithm led to the identification of six candidate heterozygous variants in three autosomal recessive IRD-associated genes: *USH2A* (M1–M4), *PDZD7* (M5) and *ADGRV1* (M6). Therefore, more than one combination of pathogenic variants was identified in this large family. Biallelic combination between the *USH2A* variants M1, M2 and M3 segregated with the disease in the third branch of the family (individuals III:17–III:23) (Fig. 1). The first branch of the family (individuals III:3–III:8) harboured also biallelic variants in *USH2A* (M2and M4), but these variants do not entirely segregate with the disease, as an unaffected sister (III:4) shared this genotype with her affected sister (III:3). Moreover, individual III:3 was also a carrier of two additional likely pathogenic variants, one in the *PDZD7* gene (M5) and another in the *ADGRV1* gene (M6), the latter being present only in the affected individual III:3. Both variants were absent in clinical databases and were classified as pathogenic and likely pathogenic according to the ACMG guidelines (Table 2). No additional candidate variant (SNVs, small indels, deep-intronic or CNVs) was identified neither in these genes nor in any of the 274 IRD genes.

## Discussion

In this study, a WGS approach was conducted to identify the genetic cause of RP in one Spanish family that remained undiagnosed despite previous studies. Currently, the use of NGS approaches in the clinical setting is primarily based on gene panels or exome analysis, both of which involve selective capturing of target regions. However, capture-based strategies have some limitations such as the lack of uniformity in terms of sequencing depth and coverage. Thus, WGS can be a better approach compared to WES as it allows not only, the identification of mutations in non-coding regions, but also, a greater sensitivity in detecting structural variants. As sequencing costs decline and bioinformatics analysis improve, WGS will have the potential to entirely replace WES [23]. Currently, filtering and prioritization of variants derived from WGS data remains challenging due to the enormous amount of information generated and the lack of systematized protocols for variant prioritization.

During this work, the optimization and implementation of a personalized diagnostic algorithm for WGS data analysis led to a reliable approach with a great versatility and high performance. The optimization process allowed minimizing the number of candidate variants pending to be validated and segregated in the available family members. This resulted in increased cost-effectiveness by reducing the amount of tedious work such as in silico predictions, manual review of the number of homozygotes in gnomAD, and Sanger sequencing. Specifically, the efficacy of the “recurrence filtering” was particularly evident, as the number of variants exclusive of one family decreased from ~ 7.2 millions to 3.9 millions. Our approach is based on an open access software and online tools which algorithms are more frequently updated compared to commercial solutions [48], facilitating data interpretation. Moreover, these filtering steps can be easily used by other researchers without investing large amount of resources in commercial licenses. In fact, despite the substantial reductions in sequencing costs, the cost of bioinformatics analyses is, in some cases, similar to sequencing; therefore, an algorithm based on free software and tools would allow the implementation of WGS in research as well as clinical practice [49].

Here, the application of the diagnostic algorithm led to the genetic diagnosis in one family which received a clinical diagnosis of RP with suspected autosomal dominant inheritance (Fig. 1). Previous analyses of the index patient (III:3) using gene-panel sequencing [16] were initially focused on the identification of variants segregating under a dominant trait but no causal mutations were detected in adRP genes. Although both *USH2A* variants (M2 and M4) were detected by the panel, the segregation was not conclusive and, therefore, WGS was conducted not only in this patient but also in two additional relatives. As a result of our hypothesis-free WGS data analysis and sequencing of more than one relative, heterozygous variants in recessive genes, *USH2A, ADGRV1* and *PDZD7*, were detected in this family. Mutations in the *USH2A* gene cause non-syndromic RP and Usher Syndrome type II, both autosomal recessive conditions [50]. In this case, three of the identified *USH2A* variants (M1, M2 and M3) were previously reported as pathogenic in ClinVar for both phenotypes, while one of them was not detected in IRD patients (M4). Remarkably, an accurate selection of the samples in which WGS is going to be conducted is highly recommended for a successful application of this pipeline.

Therefore, different combinations of *USH2A* pathogenic variants were found in this family. While individuals III:17 and III:23 harboured M3 (p.(Cys759Phe)) *in trans* with M1 (p.(His308Glnfs*16)), individual III:20 carried M3 (p.(Cys759Phe)) *in trans* with M2 (p.(Arg334Trp)). Interestingly, affected siblings (III:17, III:23 and III:20) harboured the M3 (p.(Cys759Phe)) variant in compound heterozygous status with a second variant (Fig. 1). The variant p.(Cys759Phe) is one of most prevalent *USH2A* variants, mainly associated with non-syndromic RP [51, 52]. None of the patients manifested sensorineural hearing loss, indicating that the combination of these mutations caused arRP. The expression of the phenotype varies depending on the nature of the mutations [53, 54] and these combinations results in a less severe condition, in this case, non-syndromic RP. Clinical findings also revealed the existence of intra-familial phenotypic variability among relatives of the same and different branches, reinforcing the hypothesis of more than one genetic cause underlying the phenotype in this family. In fact, affected individuals (III:3, III:17 and III:23) who are carriers of a frameshift variant manifested an earlier onset age than III:20 individual who carry two missense variants.

The index patient (III:3) harboured the *USH2A* pathogenic allele M2 (p.(Arg334Trp)) like her cousin (III:20), inherited from her affected father (II:1). However, the second mutation in *USH2A* identified in this case was the variant M4 (p.(Arg4187His)). Although this variant has been identified in the general population (6/281154 alleles in gnomAD), its frequency is consistent with disease prevalence. Computational prediction tools and conservation analyses do not provide strong support for or against an impact to the protein. Therefore, the clinical significance of the M4 is uncertain. Moreover, the segregation analysis for this variant was inconclusive, as individual III:4 was an asymptomatic carrier (aged 58 years).

Further analysis in individual III:3 led to the identification of two additional likely pathogenic variants in two genes associated with Usher Syndrome type II (*PDZD7* and *ADGRV1*). Previous studies have identified *PDZD7* variants as phenotype’s modifiers of a biallelic mutation in an USH gene, including a homozygous truncating *USH2A* mutation associated with a more severe RP when accompanied by a *PDZD7* mutation [55]. Therefore, mutations in this gene could contribute to aggravate the ocular phenotype in these cases. In addition, both *PDZD7* and *ADGRV1*, have also been associated to digenic inheritance [55]. Moreover, two pathogenic variants in two different USH2 genes (*USH2A* and *ADGRV1*) were detected in one patient suggesting that both together could be contributing to its phenotype but segregation analysis would be needed to conclude [56]. According to our proposed algorithm, the screening of mutations in deep-intronic regions in known IRD-genes must be conducted as an essential step prior to evaluate mutations in novel genes or oligogenic trait reinforcing the importance of adopting a WGS-based strategy.

Therefore, the index patient of our family (individual III:3) harboured four clinically relevant alleles: one in *PDZD7,* one in *ADGRV1* and two in *USH2A*, of which one has been reported as pathogenic while the pathogenicity of the other one remains unclear. This combination of variants is present only in this patient as it is not shared by the rest of affected individuals. One possibility would be that causative variants in the index patient remain undetected, but neither CNVs nor deep-intronic variants consistent with the disease were detected in any of the known IRD genes. Another possibility could be that the RP of individual III:3 could be caused by mutations segregating under an oligogenic inheritance among *USH2A, ADGRV1* and/or *PDZD7.* In this scenario, the variants in *PDZD7* and/or *ADGRV1* could act as genetic modifiers capable of modulating the penetrance of the milder *USH2A* allele, although further studies are needed. Oligogenic inheritance and the involvement of genetic modifiers have been demonstrated experimentally to contribute to heterogeneous disorders such as human heart diseases [57] or Bardet–Biedl syndrome [58]. Moreover, incomplete penetrance have been reported in some specific RP genes generally associated with a dominant trait [59, 60]. In addition, this mechanism has also been proposed to explain the absence of RP symptoms in other family with the homozygous *USH2A* allele p.Cys759Phe [61, 62]. In this regard, it cannot be ruled out that these two *USH2A* mutations are pathogenic but the individual III:4 has no signs of the disease due to the lack of an updated clinical evaluation, incomplete penetrance or the involvement of genetic modifiers.

The huge phenotypic overlap and genetic heterogeneity of IRDs makes that patients who received a clinical diagnosis of a particular condition may harbour causal variants in genes not specifically associated with that diagnosis. For instance, a significant number of non-syndromic RP patients can carry mutations in genes also associated with syndromic ciliopathies [63, 64]. Moreover, patients who received an initial clinical diagnosis of adRP may carry causal mutations in XLRP genes [9]. Our results are in agreement with previous studies suggesting that the contribution of mutations in recessive genes to the RP of suspected autosomal dominant pedigrees should be taken into consideration [65]. Thus, diagnostic approaches focused on a limited number of genes for a specific phenotype and mode of inheritance may not detect variants in genes not typically associated with that clinical diagnosis in a number of patients.

## Conclusions

This family is a good example of the enormous genetic and clinical heterogeneity of IRD, since within a pseudo-dominant pedigree, six different variants segregating under a recessive inheritance pattern were identified in three genes causing IRD. These results contribute to expand the mutational spectrum of IRD genes, as well as, the number of cases explained following an oligogenic inheritance. The role of genetic modifiers and oligogenic inheritance should not be underestimated in those families that remain without a conclusive genetic diagnosis, even after being thoroughly analyzed using updated approaches.

## Supplementary information

**Additional file 1: Table S1.** List of genes associated with IRD according to Retinal Information Network [5]. This list of 274 genes was used in the prioritization of variants during the application of the “IRD genes filtering”.

## Data Availability

The datasets used and/or analyzed during the current study are available from the corresponding author on reasonable request.

## Abbreviations

adRP: Autosomal dominant Retinitis Pigmentosa
arRP: Autosomal recessive Retinitis Pigmentosa
CNVs: Copy number variations
ERG: Electroretinography
gDNA: Genomic DNA
IRD: Inherited retinal dystrophies
IGV: Integrative Genomics Viewer
MAF: Minor allele frequency
NGS: Next-generation sequencing
RP: Retinitis Pigmentosa
RPE: Retinal pigment epithelium
sRP: Simplex RP
SNVs: Single nucleotide variants
VCF: Variant call format
WES: Whole exome sequencing
WGS: Whole genome sequencing
XLRP: X-linked Retinitis Pigmentosa
